# Occult Idiopathic Ventricular Fibrillation After Preoperative Cardiovascular Evaluation

**DOI:** 10.1016/j.jaccas.2026.108883

**Published:** 2026-06-18

**Authors:** Resha Reya Ganthan, Rayna Isber, Ahmed Elcicek, Joud Fahed, Nidal Isber

**Affiliations:** a Department of Internal Medicine, Richmond University Medical Center/Mount Sinai, Staten Island, New York, USA; b Department of Biology, Barnard College, Manhattan, New York, USA; c Department of Internal Medicine, Ascension Saint Agnes Hospital, Baltimore, Maryland, USA; d Department of Electrophysiology, Richmond University Medical Center/Mount Sinai, Staten Island, New York, USA

**Keywords:** electrophysiology, idiopathic ventricular fibrillation, implantable cardioverter-defibrillator, risk stratification, sudden cardiac arrest, ventricular tachycardia

## Abstract

**Background:**

Idiopathic ventricular fibrillation is characterized by malignant ventricular arrhythmias without identifiable structural heart disease or inherited arrhythmia syndrome.

**Case Summary:**

A 50-year-old man suffered out-of-hospital cardiac arrest approximately 1 hour after preoperative cardiovascular clearance for elective surgery. He had no preceding symptoms or known structural heart disease. Immediate bystander cardiopulmonary resuscitation and defibrillation resulted in successful resuscitation. Coronary angiography, cardiac magnetic resonance, electrophysiological study, and genetic testing were unrevealing. A dual-chamber implantable cardioverter-defibrillator was implanted for secondary prevention. Longitudinal device interrogation later demonstrated sustained ventricular tachycardia terminated by antitachycardia pacing.

**Discussion:**

This case highlights the limitations of current cardiovascular risk stratification in identifying occult malignant ventricular arrhythmias and reinforces the importance of implantable cardioverter-defibrillator therapy for secondary prevention.

**Take-Home Messages:**

Idiopathic ventricular fibrillation may present as sudden cardiac arrest despite unrevealing structural evaluation and apparently reassuring preoperative cardiovascular assessment. Survivors of unexplained ventricular fibrillation remain at substantial risk for recurrent malignant arrhythmias, reinforcing the importance of implantable cardioverter-defibrillator therapy for secondary prevention.

## Visual Summary

**Figure:**
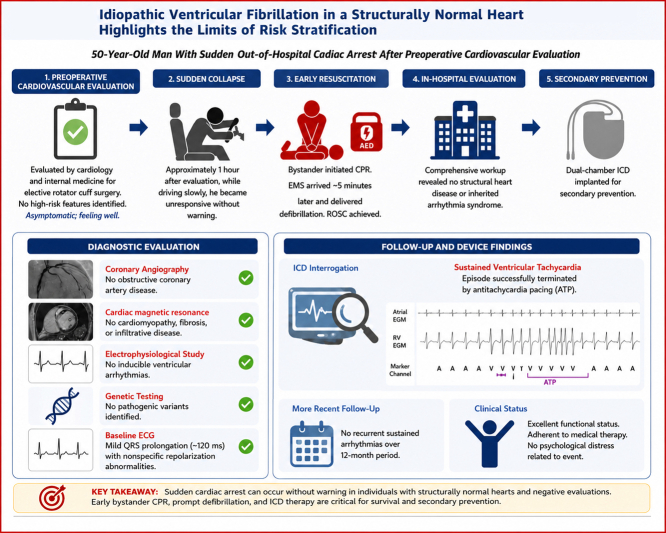

Sudden cardiac arrest (SCA) in the absence of structural heart disease represents one of the most elusive entities in cardiovascular medicine. Despite advances in imaging, electrophysiological evaluation, and genetic testing, a subset of patients present with malignant ventricular arrhythmias without an identifiable substrate, commonly referred to as idiopathic ventricular fibrillation (IVF).^1^ These cases challenge current paradigms of risk stratification, particularly when they occur in individuals recently deemed clinically stable. The inability to reliably predict such events highlights persistent gaps in contemporary cardiovascular risk assessment. Furthermore, growing recognition of concealed arrhythmogenic cardiomyopathies and evolving genotype-phenotype associations suggests that some cases previously labeled “idiopathic” may reflect occult disease processes below current diagnostic thresholds.^2^^,^^3^ We present a case of IVF occurring shortly after routine preoperative cardiovascular evaluation, emphasizing the diagnostic challenges of occult electrical disease, the limitations of current risk prediction for malignant arrhythmias in structurally normal hearts, and the critical role of secondary prevention strategies.

## History of Presentation

A 50-year-old man with a history of hypertension presented for follow-up after surviving an out-of-hospital cardiac arrest. The event occurred while he was driving with his wife, approximately 1 hour after being medically cleared by both cardiology and internal medicine for elective rotator cuff surgery. He reported feeling “the best he had felt in a long time” and denied any preceding symptoms, including chest pain, palpitations, dizziness, or syncope. Preoperative evaluation reportedly revealed no ischemic symptoms, heart failure symptoms, exertional intolerance, or known structural heart disease.

During the event, he suddenly lost consciousness, slumped over the steering wheel, and became unresponsive. His wife noted that he was unable to speak and appeared lifeless. A nearby neighbor who was a nurse initiated immediate cardiopulmonary resuscitation (CPR). Emergency medical services arrived within approximately 5 minutes and successfully defibrillated the patient. He regained spontaneous circulation but had no recollection of the event.

## Past Medical History

The patient's medical history was significant for hypertension. He had no known history of structural heart disease, prior arrhythmias, syncope, or inherited arrhythmia syndromes.

## Differential Diagnosis

Differential considerations included acute coronary syndrome, occult cardiomyopathy, inherited arrhythmia syndromes, arrhythmogenic cardiomyopathy, myocarditis, infiltrative cardiomyopathy, and primary electrical disorders including IVF.

## Investigations

**Figure 1:**
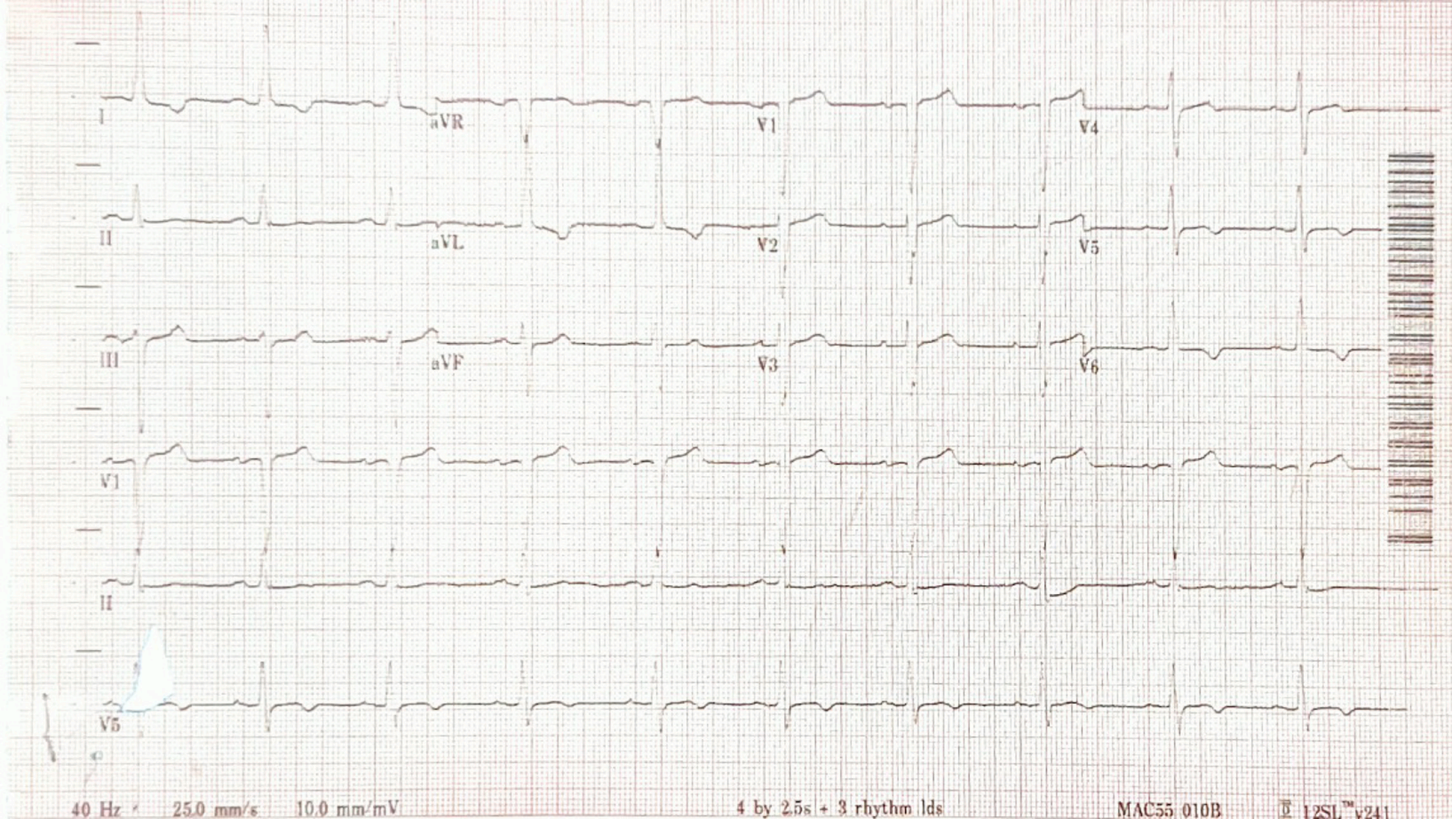
Baseline Electrocardiogram Baseline 12-lead electrocardiogram demonstrating sinus rhythm with mild QRS prolongation (approximately 120 milliseconds), left ventricular hypertrophy, and nonspecific repolarization abnormalities without definitive evidence of an inherited arrhythmia syndrome.

Comprehensive cardiovascular evaluation was performed. Coronary angiography revealed no obstructive coronary artery disease. Cardiac magnetic resonance demonstrated no evidence of cardiomyopathy, myocardial fibrosis, or infiltrative disease. Baseline electrocardiography demonstrated sinus rhythm with mild QRS prolongation (approximately 120 milliseconds), left ventricular hypertrophy, and nonspecific repolarization abnormalities without definitive evidence of an inherited arrhythmia syndrome (Figure 1).

**Figure 2:**
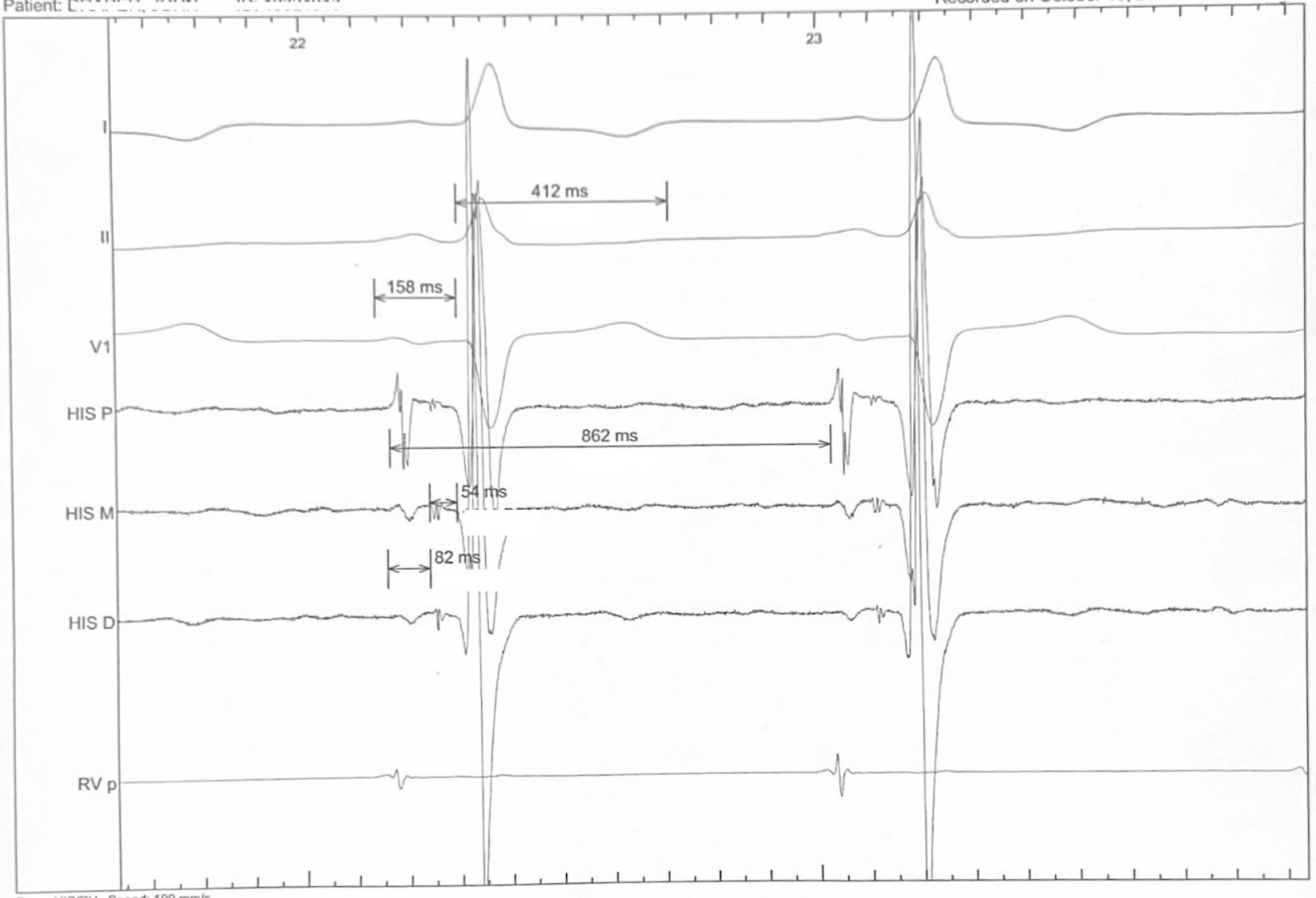
Electrophysiology Study With His Bundle Recordings Intracardiac recordings obtained during an electrophysiological study demonstrating normal sinus node function and preserved atrioventricular nodal and infra-Hisian conduction properties, including an AH interval of 82 milliseconds and an HV interval of 54 milliseconds. No bypass tract, dual atrioventricular nodal physiology, supraventricular tachycardia, or sustained ventricular tachyarrhythmias were induced during programmed stimulation, supporting the diagnosis of idiopathic ventricular fibrillation after exclusion of identifiable arrhythmogenic substrates. AH = atrio-His interval; HV = His-ventricular interval.

An electrophysiological study demonstrated normal sinus node function with normal atrioventricular nodal and infra-Hisian conduction properties (Figure 2). Baseline conduction intervals were preserved, including an atrio-His interval of 82 milliseconds and His-ventricular interval of 54 milliseconds. No evidence of a bypass tract or dual atrioventricular nodal physiology was identified. Programmed electrical stimulation failed to induce supraventricular tachycardia or sustained ventricular tachyarrhythmias. The absence of inducible ventricular arrhythmias during programmed stimulation further emphasized the diagnostic uncertainty surrounding the patient's presentation and highlighted the limitations of current electrophysiologic testing in excluding future malignant ventricular arrhythmias.

Genetic testing for inherited arrhythmia syndromes was negative. Family screening with electrocardiograms and echocardiography in his children was unremarkable.

## Management

A dual-chamber implantable cardioverter-defibrillator (ICD) was inserted for secondary prevention. The patient was treated with aspirin, diltiazem, metoprolol, atorvastatin, losartan, omeprazole, and inhaled fluticasone.

## Outcome and Follow-Up

At follow-up, the patient reported excellent functional status and adherence to medical therapy. Interestingly, he reported minimal psychological distress related to the event, largely because he had no recollection of the arrest itself.

**Figure 3:**
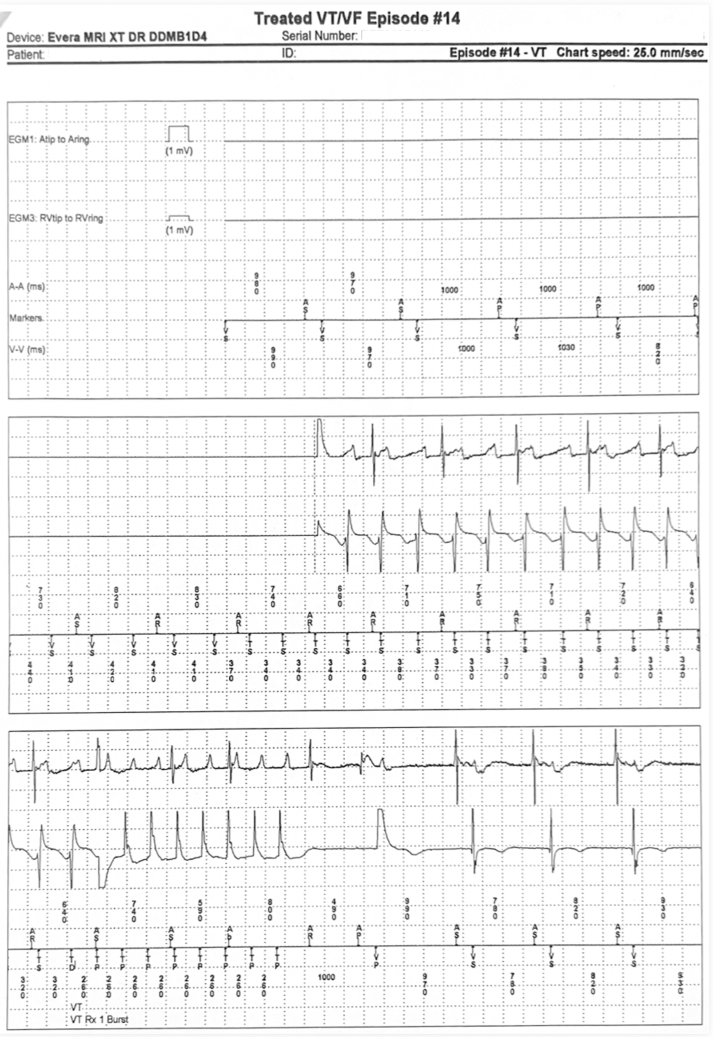
Implantable Cardioverter-Defibrillator Electrogram Demonstrating Sustained Ventricular Tachycardia Device interrogation electrogram demonstrating a sustained monomorphic ventricular tachycardia episode with a cycle length of approximately 340 milliseconds successfully terminated with antitachycardia pacing, confirming persistent arrhythmogenic risk despite a structurally normal heart and unrevealing diagnostic evaluation. VF = ventricular fibrillation; VT = ventricular tachycardia.

Device interrogation demonstrated normal function with appropriate sensing and pacing parameters. During longitudinal follow-up, the ICD recorded episodes of ventricular tachycardia, including one sustained monomorphic ventricular tachycardia episode with a cycle length of approximately 340 milliseconds successfully terminated by antitachycardia pacing, confirming persistent arrhythmogenic risk despite otherwise unrevealing diagnostic evaluation (Figure 3). More recent interrogation showed no recurrent sustained arrhythmias over a 12-month period.

## Discussion

This case highlights several critical aspects of IVF and the limitations of current cardiovascular risk stratification.

First, the temporal relationship between recent preoperative cardiovascular evaluation and SCA highlights the inherent limitations of current perioperative risk stratification paradigms in identifying occult malignant ventricular arrhythmias. Contemporary preoperative cardiovascular assessment is primarily designed to estimate ischemic risk, heart failure risk, and perioperative hemodynamic tolerance rather than predict IVF in patients without structural heart disease or recognized inherited arrhythmia syndromes.^1^ Importantly, this case should not be interpreted as a failure of guideline-directed evaluation, as the patient lacked conventional high-risk clinical features or identifiable structural abnormalities. Rather, it emphasizes that SCA may occasionally represent the first manifestation of concealed electrical disease despite otherwise appropriate cardiovascular assessment.

Current perioperative cardiovascular evaluation frameworks rely heavily on symptom burden, functional capacity, ischemic heart disease, ventricular function, and procedural risk estimation. However, these models are not intended to detect occult primary electrical disorders in asymptomatic individuals with structurally normal hearts. This distinction is clinically important when interpreting rare catastrophic arrhythmic events occurring despite apparently reassuring preoperative evaluation.^7^

Second, despite a comprehensive negative work-up—including normal coronary angiography, cardiac magnetic resonance, noninducible electrophysiological study, and negative genetic testing—the patient demonstrated subtle baseline electrical abnormalities and recurrent ventricular arrhythmias on ICD follow-up. The baseline electrocardiogram demonstrated mild QRS prolongation, left ventricular hypertrophy, and nonspecific repolarization abnormalities, although these findings were not diagnostic of a specific inherited arrhythmia syndrome.^4^ Furthermore, despite a noninducible electrophysiological study, longitudinal device interrogation later demonstrated sustained monomorphic ventricular tachycardia successfully terminated by antitachycardia pacing, confirming persistent arrhythmogenic susceptibility. This supports the concept that so-called idiopathic ventricular fibrillation (VF) may often represent undetected substrates below current diagnostic thresholds.^5^ Advanced imaging modalities such as cardiac magnetic resonance have high sensitivity for cardiomyopathies, including hypertrophic cardiomyopathy; however, microstructural abnormalities, early fibrosis, or localized myocardial disarray may remain beyond the resolution of current imaging modalities.^2^

Third, evolving genetic understanding suggests that idiopathic VF is increasingly a provisional diagnosis. Although initial genetic testing was negative, newer genes associated with arrhythmogenic risk—including FLNC, PLN, LMNA, and DSP—have been identified in recent years.^3^^,^^6^ In addition, polygenic risk and noncoding genomic variants may contribute to arrhythmogenesis, further complicating the diagnostic landscape. These findings support the concept that so-called idiopathic VF may reflect concealed arrhythmogenic substrates below current diagnostic thresholds.

Fourth, this case reinforces the indispensable role of ICD therapy in secondary prevention. Patients with unexplained cardiac arrest have a high recurrence risk, and ICD therapy significantly improves survival. In contemporary cohorts, appropriate ICD therapies occur in up to 30% to 40% of secondary prevention patients over follow-up.^7^ The documented sustained ventricular tachycardia in this patient further validates the decision for device implantation.

Fifth, the patient's survival was critically dependent on immediate bystander CPR and rapid defibrillation, emphasizing an important public health message. Early CPR and defibrillation remain the strongest determinants of neurologically intact survival after out-of-hospital cardiac arrest.^8^ This case illustrates that, although risk prediction remains imperfect, outcomes can be dramatically improved through community preparedness.

Finally, the absence of psychological distress due to amnesia of the event represents an unusual but insightful aspect of survivorship. Although many survivors experience anxiety and post-traumatic stress, this patient's perception highlights variability in psychological adaptation after SCA.

## Conclusions

This case illustrates the diagnostic challenges associated with occult malignant ventricular arrhythmias in patients without apparent structural heart disease or identifiable inherited arrhythmia syndromes. Despite comprehensive negative evaluation, the presence of recurrent ventricular arrhythmias confirms an underlying arrhythmogenic substrate. The case underscores the evolving role of genetic testing, the importance of longitudinal follow-up, and the lifesaving impact of ICD therapy. Furthermore, it highlights the critical role of early bystander CPR in survival. Continued advances in electrophysiologic characterization, high-resolution imaging, genomics, and longitudinal arrhythmia phenotyping may improve future identification of concealed arrhythmogenic substrates currently below diagnostic thresholds.

**Take-Home Messages**

• Idiopathic ventricular fibrillation may present as sudden cardiac arrest despite unrevealing structural evaluation and apparently reassuring preoperative cardiovascular assessment.
• Survivors of unexplained ventricular fibrillation remain at substantial risk for recurrent malignant arrhythmias, reinforcing the importance of implantable cardioverter-defibrillator therapy for secondary prevention.

**Visual Summary:**
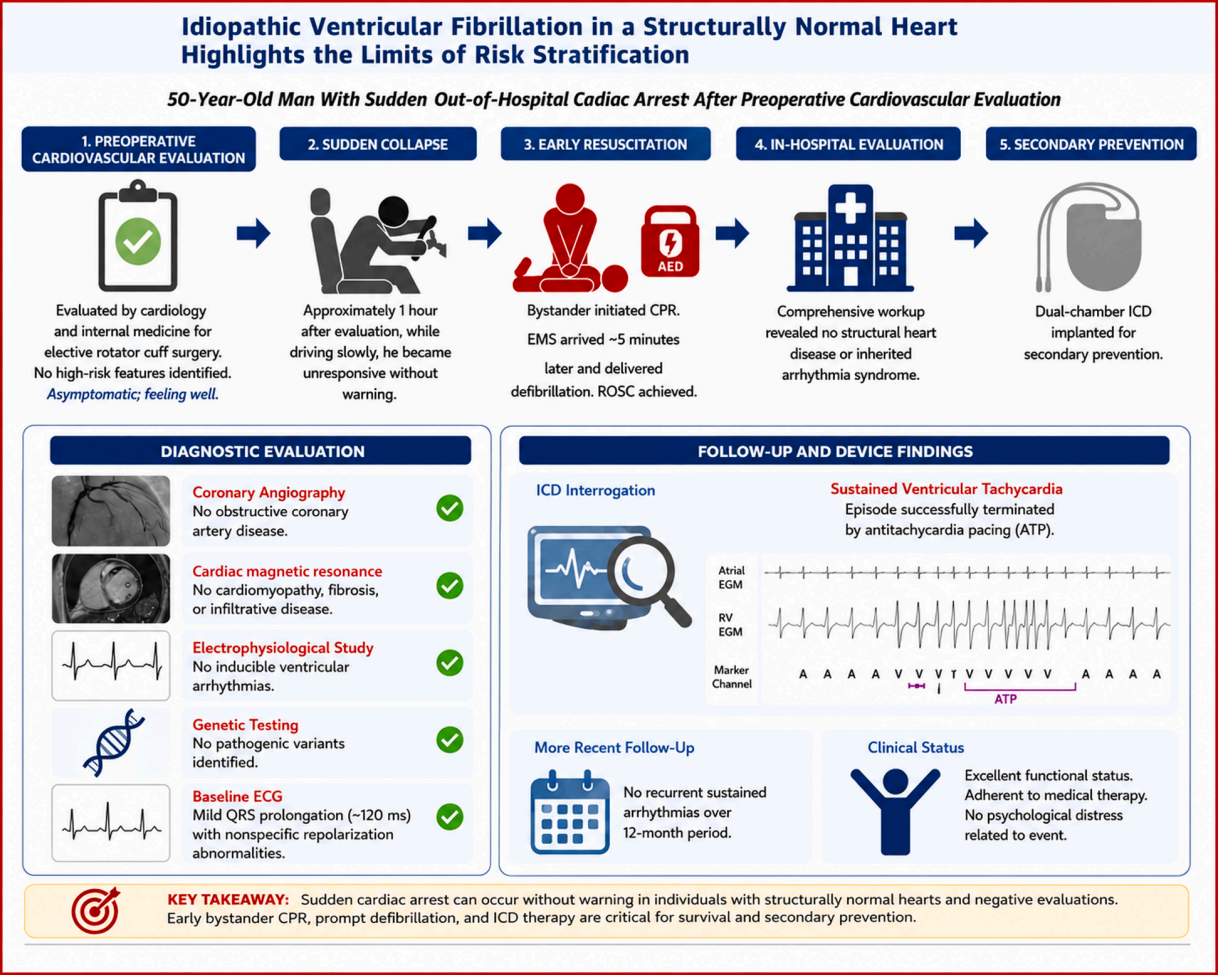
Occult Idiopathic Ventricular Fibrillation Presenting as Sudden Out-of-Hospital Cardiac Arrest Shortly After Preoperative Cardiovascular Evaluation in a Structurally Normal Heart Comprehensive diagnostic evaluation was unrevealing; however, longitudinal implantable cardioverter-defibrillator follow-up demonstrated sustained ventricular tachycardia terminated by antitachycardia pacing, supporting persistent arrhythmogenic risk and the importance of secondary prevention. AED = automated external defibrillator; ATP = antitachycardia pacing; CPR = cardiopulmonary resuscitation; ECG = electrocardiogram; EGM = electrogram; EMS = emergency medical services; ICD = implantable cardioverter-defibrillator; ROSC = return of spontaneous circulation; RV = right ventricular.

## Funding Support and Author Disclosures

The authors have reported that they have no relationships relevant to the contents of this paper to disclose.

## References

[1] Priori S.G., Blomström-Lundqvist C., Mazzanti A. (2022). 2022 ESC guidelines for the management of patients with ventricular arrhythmias and prevention of sudden cardiac death. Eur Heart J.

[2] Maron B.J., Rowin E.J., Maron M.S. (2022). Diagnosis and evaluation of hypertrophic cardiomyopathy. J Am Coll Cardiol.

[3] Towbin J.A., McKenna W.J., Abrams D.J. (2019). 2019 HRS expert consensus statement on evaluation, risk stratification, and management of arrhythmogenic cardiomyopathy. Heart Rhythm.

[4] Viskin S., Rosso R., Rogowski O. (2012). The “short-coupled” variant of idiopathic ventricular fibrillation. J Am Coll Cardiol.

[5] Haïssaguerre M., Shoda M., Jaïs P. (2002). Mapping and ablation of idiopathic ventricular fibrillation. Circulation.

[6] Towbin J.A., McKenna W.J., Abrams D.J. (2019). 2019 HRS expert consensus on evaluation of cardiomyopathy. Heart Rhythm.

[7] Bhave N.M., Cibotti-Sun M., Moore M.M. (2024). 2024 ACC/AHA guideline for perioperative cardiovascular management for noncardiac surgery. J Am Coll Cardiol.

[8] Perkins G.D., Graesner J.T., Semeraro F. (2021). European resuscitation council guidelines 2021: epidemiology of cardiac arrest and systems saving lives. Resuscitation.

